# Assessment of MGMT promoter methylation status in glioblastoma using deep learning features from multi-sequence MRI of intratumoral and peritumoral regions

**DOI:** 10.1186/s40644-024-00817-1

**Published:** 2024-12-23

**Authors:** Xuan Yu, Jing Zhou, Yaping Wu, Yan Bai, Nan Meng, Qingxia Wu, Shuting Jin, Huanhuan Liu, Panlong Li, Meiyun Wang

**Affiliations:** 1 Department of Radiology, Henan Provincial People’s Hospital & the People’s Hospital of Zhengzhou University, 7 Weiwu Road, Zhengzhou, 450000 PR China; 2 Biomedical Research Institute, Henan Academy of Sciences, Zhengzhou, China; 3 Key Laboratory of Science and Engineering for the Multi-modal Prevention and Control of Major Chronic Diseases, Ministry of Industry and Information Technology, Zhengzhou, China; 4 School of Computer Science and Technology, Wuhan University of Science and Technology, Wuhan, China

**Keywords:** Glioblastoma, O^6^-methylguanine-DNA methyltransferase, Magnetic resonance imaging, Deep learning

## Abstract

**Objective:**

This study aims to evaluate the effectiveness of deep learning features derived from multi-sequence magnetic resonance imaging (MRI) in determining the O^6^-methylguanine-DNA methyltransferase (MGMT) promoter methylation status among glioblastoma patients.

**Methods:**

Clinical, pathological, and MRI data of 356 glioblastoma patients (251 methylated, 105 unmethylated) were retrospectively examined from the public dataset The Cancer Imaging Archive. Each patient underwent preoperative multi-sequence brain MRI scans, which included T1-weighted imaging (T1WI) and contrast-enhanced T1-weighted imaging (CE-T1WI). Regions of interest (ROIs) were delineated to identify the necrotic tumor core (NCR), enhancing tumor (ET), and peritumoral edema (PED). The ET and NCR regions were categorized as intratumoral ROIs, whereas the PED region was categorized as peritumoral ROIs. Predictive models were developed using the Transformer algorithm based on intratumoral, peritumoral, and combined MRI features. The area under the receiver operating characteristic curve (AUC) was employed to assess predictive performance.

**Results:**

The ROI-based models of intratumoral and peritumoral regions, utilizing deep learning algorithms on multi-sequence MRI, were capable of predicting MGMT promoter methylation status in glioblastoma patients. The combined model of intratumoral and peritumoral regions exhibited superior diagnostic performance relative to individual models, achieving an AUC of 0.923 (95% confidence interval [CI]: 0.890 – 0.948) in stratified cross-validation, with sensitivity and specificity of 86.45% and 87.62%, respectively.

**Conclusion:**

The deep learning model based on MRI data can effectively distinguish between glioblastoma patients with and without MGMT promoter methylation.

## Key Points

1. The MRI peritumoral features have a beneficial effect on the diagnosis of MGMT promoter methylation status in glioblastoma.

2. The combination of intratumoral and peritumoral T1WI and CE-T1WI MRI features has the optimal diagnostic accuracy for evaluating the methylation status of MGMT promoter in glioblastoma.

3. The developed Transformer deep learning model improved diagnostic efficacy of MGMT promoter methylation status.

## Introduction

Glioblastoma ranks among the most invasive brain tumors, characterized by a high degree of malignancy [1, 2]. Despite progress in medical treatments such as surgery, radiotherapy, and chemotherapy, the prognosis for glioblastoma patients remains dismal [3]. Temozolomide (TMZ), a standard component of glioblastoma treatment regimens, shows therapeutic efficacy closely linked to the methylation status of O^6^-methylguanine-DNA methyltransferase (MGMT) [4, 5]. MGMT, a DNA repair enzyme, experiences inhibited DNA repair activity when its promoter is methylated, rendering tumor cells more susceptible to the cytotoxic effects of TMZ [6]. Therefore, MGMT promoter methylation level serves as a crucial indicator of the effectiveness of alkylating agents in controlling glioblastoma cells [7]. The primary method for determining MGMT promoter methylation status currently involves surgical sampling, followed by detection via methylation-specific polymerase chain reaction (PCR), pyrosequencing (both detecting the MGMT promoter region directly), or immunohistochemistry (identifying MGMT protein expression) [4]. However, these methods are time-consuming and pose potential risks of neurological damage during biopsy. Given the highly heterogeneous nature of glioblastoma, pathological tissue obtained via biopsy may not fully represent the tumor’s biological characteristics. Magnetic resonance imaging (MRI) effectively illustrates the invasive extent and mass effect of glioblastoma, making it the preferred imaging modality for its examination [8–10]. Research has indicated that imaging features of the tumor microenvironment provide additional valuable insights into the tumor’s biological characteristics [11, 12]. Hence, this study aims to investigate the efficacy of assessing MGMT promoter methylation status through intratumoral and peritumoral imaging features derived from multiparametric MRI.

In recent years, artificial intelligence (AI) has emerged as a novel non-invasive approach for tumor research [13, 14]. By establishing associations between imaging data and clinical data, AI has enhanced the precision of tumor diagnosis and treatment, demonstrating extensive potential in guiding clinical decision-making [15]. The Transformer algorithm, a deep learning model relevant to brain tumor diagnosis and treatment, has garnered significant attention from researchers [16]. The Transformer model employs attention mechanisms to accelerate training speed, enabling effective processing and analysis. Studies have indicated that Transformers are pivotal in brain tumor MRI analysis, with substantial implications for pathological grading based on MRI and tumor tissue sections, prediction of brain tumor molecular expression, and forecasting brain tumor radiotherapy outcomes [17–19]. Consequently, this study intends to utilize the Transformer algorithm to extract intratumoral and peritumoral imaging features from MRI and develop a predictive model for assessing MGMT promoter methylation status in glioblastoma patients, aiming to offer crucial informational guidance for related clinical diagnosis and treatment.

## Materials and methods

### Patient information

**Fig. 1:**
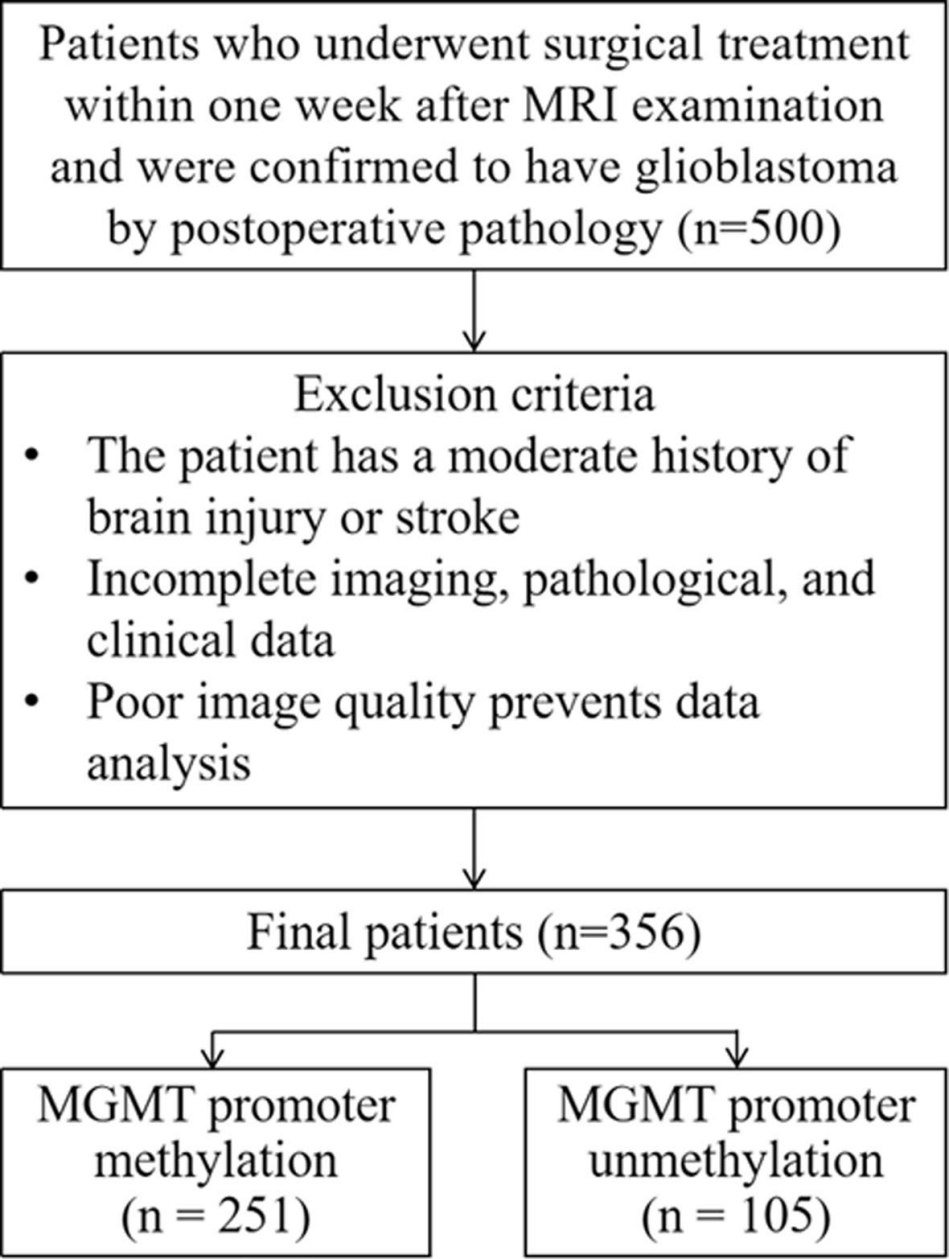
Flow diagram of the patient selection process

This retrospective study was conducted in accordance with the principles of the Declaration of Helsinki, utilizing case data from The Cancer Imaging Archive (TCIA) [20, 21]. The inclusion criteria included: (1) comprehensive imaging, pathological, and clinical data; (2) pathological confirmation of glioblastoma (WHO CNS 2021) with a definitive diagnosis of MGMT promoter methylation status (in house method developed by UCSF clinical labs, https://genomics.ucsf.edu/content/mgmt-promoter-methylation-assay); (3) surgical intervention within one week following MRI examination; and (4) absence of any prior surgical, radiotherapy, or chemotherapy treatments before the MRI examination. The exclusion criterion was defined as poor visualization of lesions in MRI images, impeding data analysis. Consequently, 356 patients were incorporated into the study, with relevant clinical information, such as patient gender and age, documented (Fig. 1).

### Inspection methods

All preoperative MRI was performed on the same model of 3.0 T scanner (Discovery 750, GE Healthcare, Waukesha, Wisconsin, USA) and a dedicated 8-channel head coil (Invivo, Gainesville, Florida, USA). The imaging protocol included T1-weighted imaging (T1WI) and contrast-enhanced T1-weighted imaging (CE-T1WI). All tumors were tested for MGMT methylation status using a methylation sensitive quantitative PCR assay.

### Image segmentation

**Fig. 2:**
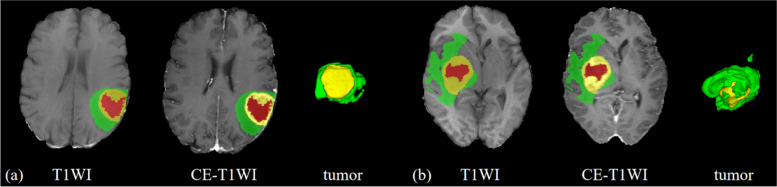
Segmentation illustration of tumor T1WI and CE-T1WI sequence imaging. Patient a is a 66-year-old male with unmethylated MGMT status, and patient b is a 68-year-old female with methylated MGMT status. The red region represents the NCR, the yellow region represents the ET, and the green region represents the PED

The T1WI and CE-T1WI sequences were imported into ITK-SNAP software (Version 3.60, http://www.itk-snap.org). Initially, all lesions were segmented automatically utilizing a segmentation algorithm focusing on the region of interest (ROI) of the entire primary lesion. Subsequently, these segmentation outcomes were manually refined by a radiologist with five years of experience, alongside an associate chief radiologist with 12 years of experience. Figure 2 illustrates the lesion segmentation process. The segmented areas encompassed the necrotic tumor core (NCR), enhancing tumor (ET), and peritumoral edema (PED). In this investigation, the segmented ET and NCR regions were categorized as intratumoral ROI, whereas the PED region was classified as peritumoral ROI.

### Development of Transformer deep learning model

#### Image data preprocessing

Initially, image data are preprocessed to achieve a standardized format for the model. T1WI and CE-T1WI sequences are co-registered using FSL software (Version 5.0, https://fsl.fmrib.ox.ac.uk) and uniformly resampled to an isotropic resolution of 1 mm^3^. Utilizing the SPM tool (https://www.fil.ion.ucl.ac.uk/spm/), skull-stripping and random axial mirroring are executed according to a standard head MRI data template. Subsequently, image normalization is performed, involving cropping to a uniform size and rescaling image intensity to a range of 0 – 1 [22].

#### Saliency-aware enhancement of ROI

Image data and ROI are input into the model simultaneously. Since the tumor often occupies a small area in a brain MRI, a saliency-aware method is introduced to emphasize the tumor ROI to allow the model to focus on the intratumoral ROIs or peritumoral ROIs. Based on tumor masks, the intensity values of pixels in the non-tumor regions are scaled down by a certain factor (for example, 1/3 of their original values in our tests). Consequently, the significant tumor regions are highlighted.

#### Data partitioning

Exploiting the characteristics of the Transformer, input glioblastoma images are segmented into multiple patches, each constituting a fixed-size square region.

#### Embedding layer

Each patch undergoes transformation into a fixed-dimensional vector through linear transformation, a process referred to as patch embedding, to facilitate subsequent model computations.

#### Positional encoding

To retain the positional information of patches within the image, positional encoding is incorporated into each patch, aiding the model's comprehension of spatial relationships within the image.

#### Transformer encoder

An encoder, structured with a series of Transformers, is employed to process the embedded vectors. The input data are subjected to interaction and transformation through multi-head self-attention (MSA) mechanisms and multilayer perceptron (MLP) structures.

#### Decoder

Post-encoding, the data are relayed to the decoder via skip connections, progressively generating deep learning features.

#### Feature fusion

The extracted deep learning image features are integrated with clinical features, and the predictive outcomes for glioblastoma MGMT promoter methylation status are generated through fully connected layers.

#### Training and optimization

Model training and optimization are continuously executed throughout the process via backpropagation and optimization functions, enhancing its efficacy in predicting glioblastoma MGMT promoter methylation status.

**Fig. 3:**
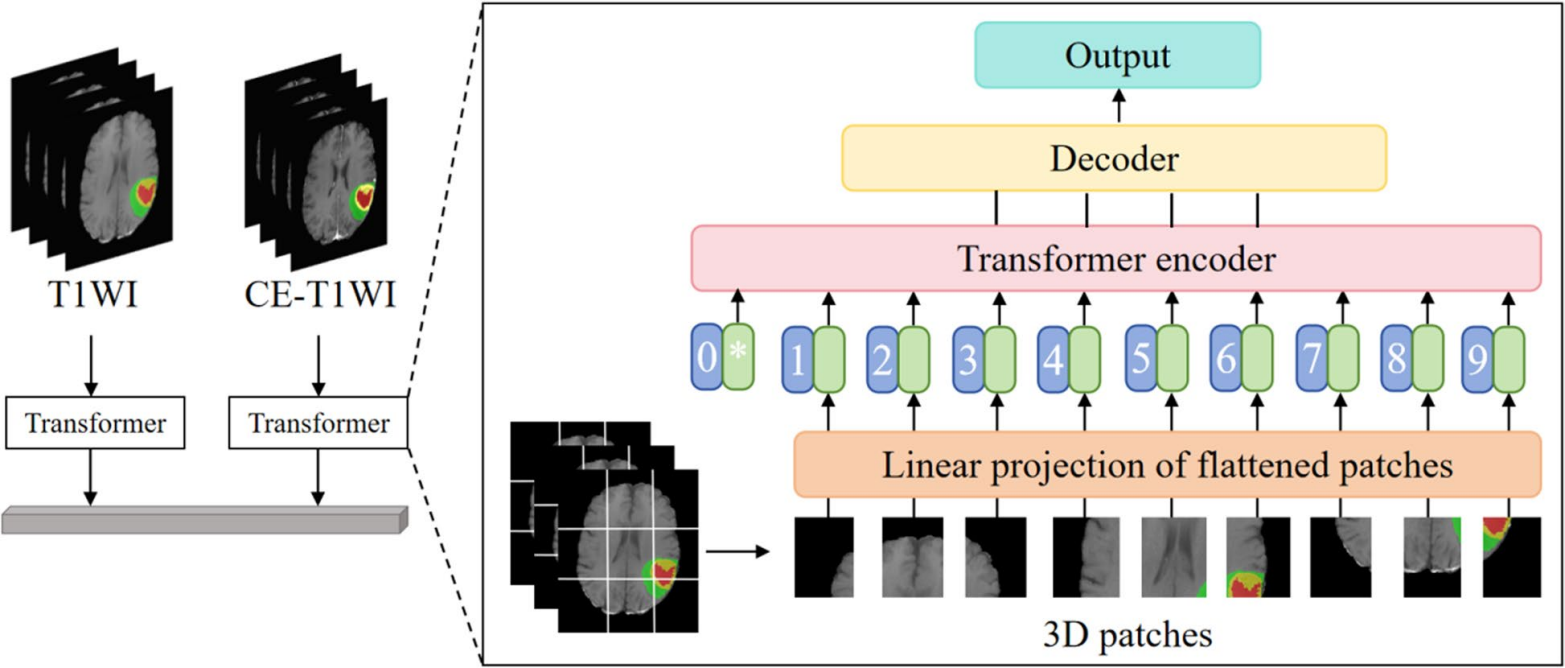
Schematic diagram illustrating the details of the Transformer model used in this study

The schematic diagram of the Transformer model are shown in Fig. 3. The optimization details for the Transformer model in this study are as follows:

In step (c) mentioned above, whereas conventional natural language processing employs a Transformer for one-dimensional input encoded sequences, this research considers glioblastoma imaging data as three-dimensional input voxels characterized by dimensions $x\in {R}^{H\times W\times D\times C}$ (where $H$, $W$, and $D$ represent the resolution, and $C$ represents the number of input channels). These voxels are partitioned into flattened, uniform, non-overlapping patches. Each patch has a resolution of $P$, resulting in a sequence length of $N=(H\times W\times D)/{P}^{3}$.

In steps (d) and (e) mentioned above, all patches are projected into a K-dimensional embedding space via a linear layer, maintaining consistency across the Transformer layers. To retain the spatial feature information of each extracted patch, a one-dimensional learnable positional embedding ${E}_{pos}\in {R}^{N\times K}$ is incorporated into the projected patch embedding $E\in {R}^{({P}^{3}\cdot C)\times K}$ using the following method:

1 $${z}_{0}=[{x}_{v}^{1}E;{x}_{v}^{2}E;...;{x}_{v}^{N}E]{+E}_{pos}$$

In step (f) mentioned above, the transformer blocks, encompassing MSA and MLP sublayers, are stacked using the following formula:

2 $${z^{\prime}}_{i}=MSA(Norm({z}_{i-1}))+{z}_{i-1}, i=1...L$$

3 $${z}_{i}=MLP(Norm({z^{\prime}}_{i}))+{z^{\prime}}_{i}, i=1...L$$

where $\text{Norm}()$ represents the normalization layer, while MLP consists of two linear layers with GELU activation functions. $i$ represents the identifier of the intermediate patch, and $L$ represents the number of Transformer layers.

An MSA sublayer comprises $n$ parallel self-attention (SA) mechanisms. SA is a parameterized function that learns the mapping relationships between the query (q) matrix, corresponding key (k) matrix, and value (v) matrix of sequence $z\in {R}^{N\times K}$. The SA weight matrix $A$ is computed by evaluating the similarity between key-value pairs in $z$ using the following formula:

4 $$A=softmax(\frac{q{k}^{T}}{\sqrt{{K}_{h}}})$$

where ${K}_{h}=\frac{K}{n}$ represents a scaling factor that maintains a constant parameter count when selecting different k-values. The calculated attention weight matrix is used to compute the SA output by weighting the values in the sequence as follows:

5 $$SA(z)=Av$$

where $v$ represents the values of the input sequence. Furthermore, the output of MSA is defined as:

6 $$MSA(z)=[{SA}_{1}(z);{SA}_{2}(z);...;{SA}_{n}(z)]{W}_{msa}$$

${W}_{msa}\in {R}^{n.{k}_{h}\times k}$ represents the multi-head trainable parameter weights.

In step (g) mentioned above, the multi-resolution features of the encoder are integrated with the decoder to extract a sequence representation ${z}_{i}(i\in \{\text{3,6},\text{9,12}\})$ with dimensions $\frac{H\times W\times D}{{P}^{3}}\times K$. This representation is then transformed into a tensor of size $\frac{H}{P}\times \frac{W}{P}\times \frac{D}{P}\times K$ through a Transformer. At each resolution level, the reconstructed tensor is projected from the embedding space to the input space through consecutive $3\times 3\times 3$ convolutional layers and normalization layers.

Within this bottleneck structure encoder, the transformed feature maps undergo deconvolution, effectively doubling their resolution. The upsampled feature maps are then concatenated with those produced by the Transformer. These combined features are subsequently input into consecutive $3\times 3\times 3$ convolutional layers, followed by a deconvolution layer for further upsampling. This sequence is iteratively repeated for subsequent layers until the original resolution is achieved. Ultimately, the output is fed into a $1\times 1\times 1$ convolutional layer with a softmax activation function, generating the glioblastoma MGMT promoter methylation status results.

In step (h) mentioned above, due to the imbalanced dataset in this classification problem, where the ratio of MGMT promoter methylated to unmethylated samples is approximately 2.5:1, a dynamic focal loss function was implemented. A modulating factor was introduced to diminish the weight of easily classified samples, thus enabling the model to prioritize difficult-to-classify samples during training. Throughout model training, the loss contributions from different classes were independently rebalanced based on the imbalance degree between positive and negative samples within each class. The loss was dynamically adjusted according to the training status of various classes to address the sample imbalance issue, thereby enhancing model performance and achieving accurate prediction results.

7 $$DFL({p}_{t})=-{{\alpha }_{t}(1-{p}_{t})}^{{\gamma }^{j}}log({p}_{t})$$

The likelihood of a sample being predicted as methylated by the model is represented as ${p}_{t}$, while ${\alpha }_{t}$ and ${\gamma }^{j}$ represent pivotal hyperparameters balancing positive and negative samples.

**Fig. 4:**
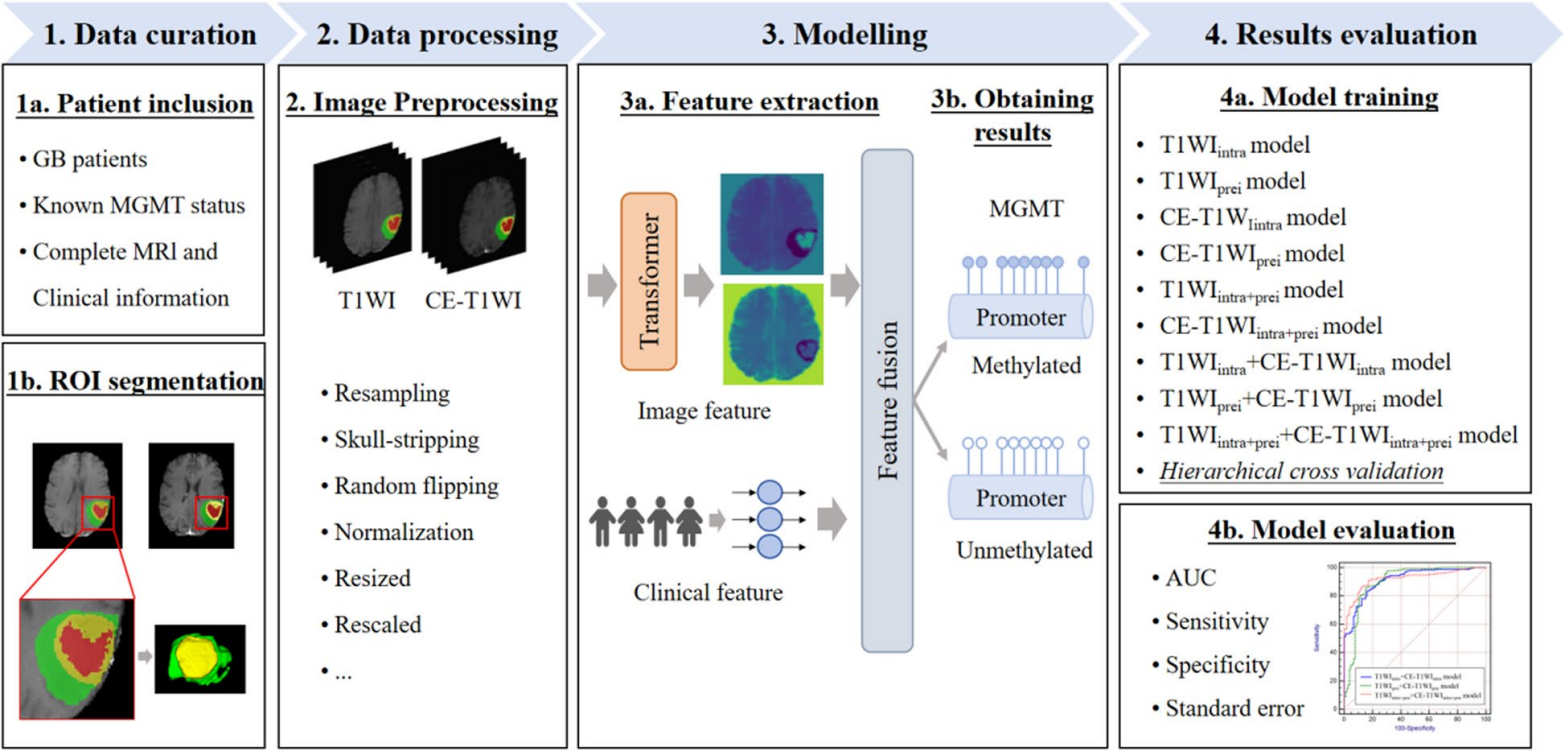
Schematic diagram of the research process

Schematic diagram of the research process is shown in Fig. 4. The performance of each classifier was ultimately assessed by computing the average values of the AUC, sensitivity, specificity, and standard error derived from validation.

### Statistical analysis

To compare categorical data between groups, Chi-square tests were applied, whereas Mann–Whitney U tests or independent samples *t*-tests were utilized for continuous data comparisons. The performance of the predictive models was assessed by evaluating AUC, sensitivity, specificity, and standard error. All statistical analyses were performed using R software (Version 4.3.3). A *P* < 0.05 was deemed statistically significant. Institutional Review Board approval was obtained.

## Results

### Statistical analysis of clinical features

In this study, 251 patients with MGMT promoter methylation and 105 patients without MGMT methylation were included. A significant difference in gender distribution between the two groups was found (Table 1). Therefore, gender was included as a clinical indicator in the model.

**Table 1:** Statistical analysis of clinical features

Characteristic	Methylation (*n* = 251)	Unmethylation (*n* = 105)	z/Chi-Square	*P* value
Age (years)	59.26 ± 13.77	59.376 ± 13.72	−0.747	0.455
Gender			6.671	0.010
Male	140 (55.78%)	74 (70.48%)		
Female	111 (44.22%)	31 (29.52%)		

### Model construction

Clinical information was integrated with intratumoral and peritumoral data from T1WI and CE-T1WI sequences and input into the Transformer algorithm to sequentially construct nine models. These models comprised four single-sequence models (T1WI_intra_ model, T1WI_peri_ model, CE-T1WI_intra_ model, and CE-T1WI_peri_ model), two single-sequence combined intratumoral and peritumoral models (T1WI_intra+peri_ model and CE-T1WI_intra+peri_ model), two multi-sequence models (T1WI_intra_ model + CE-T1WI_intra_ model and T1WI_peri_ + CE-T1WI_peri_ model), and one multi-sequence combined intratumoral and peritumoral model (T1WI_intra+peri_ + CE-T1WI_intra+peri_ model).

### Predictive performance

Stratified cross-validation was utilized in this study for model training and validation. The generalization ability of each model was evaluated through five-fold data partitioning, training, and testing, ensuring that the ratio of MGMT promoter methylated to unmethylated samples in each training and test set closely mirrored that of the entire dataset, thereby maintaining data representativeness throughout the cross-validation process. The results (Table 2) indicated that both intratumoral and peritumoral models of T1WI and CE-T1WI were markedly correlated with MGMT promoter methylation status. When using single-sequence MRI, the CE-T1WI_intra_ model exhibited the best performance in predicting MGMT promoter methylation status (AUC: 0.867, Sensitivity: 83.67%, Specificity: 80.95%, SE: 0.0192). Regardless of intratumoral or peritumoral models, CE-T1WI outperformed T1WI in prediction results, and the AUC differences between T1WI and CE-T1WI models were not statistically significant (T1WI_intra_ model vs. CE-T1WI_intra_ model: Z = 0.210, *P* = 0.8339; T1WI_peri_ model vs. CE-T1WI_peri_ model: Z = 0.0602, *P* = 0.9520). As shown in Table 2, combined models (T1WI_intra+peri_ model and CE-T1WI_intra+peri_ model) demonstrated enhanced diagnostic performance compared to individual intratumoral or peritumoral models, with no significant difference in AUC between T1WI_intra+peri_ model and CE-T1WI_intra+peri_ model (Z = 0.539, *P* = 0.5901).

**Table 2:** Prediction results of MGMT promoter methylation status using different models

	AUC (95% confidence interval)	Sensitivity (%)	Specificity (%)	Standard error
Single-sequence models				
T1WI_intra_ model	0.861 (0.820—0.895)	82.47	76.19	0.0228
T1WI_prei_ model	0.850 (0.809—0.886)	76.49	78.10	0.0229
CE-T1WI_intra_ model	0.867 (0.828—0.901)	83.67	80.95	0.0192
CE-T1WI_prei_ model	0.852 (0.811—0.887)	79.28	81.90	0.0205
Two single-sequence combined intratumoral and peritumoral models				
T1WI_intra+prei_ model	0.874 (0.631—0.865)	75.30	81.90	0.0206
CE-T1WI_intra+prei_ model	0.889 (0.851—0.920)	85.26	80.95	0.0177
Multi-sequence models				
T1WI_intra_ + CE-T1WI_intra_ model	0.914 (0.879—0.941)	83.67	83.81	0.0155
T1WI_prei_ + CE-T1WI_prei_ model	0.901 (0.865—0.930)	86.06	84.76	0.0215
Multi-sequence combined intratumoral and peritumoral model				
T1WI_intra+prei_ + CE-T1WI_intra+prei_ model	0.923 (0.890—0.948)	86.45	87.62	0.0141

*MGMT* O^6^-methylguanine-DNA methyltransferase, *AUC* area under the receiver operating characteristic curve, *T1WI* T1-weighted imaging, *CE-T1WI* contrast-enhanced T1-weighted imaging, *intra* intratumoral region, *prei* peritumoral region

In comparison to single-sequence MRI models, the multi-sequence combined MRI models showed enhanced diagnostic performance. The T1WI_intra_ + CE-T1WI_intra_ model, T1WI_peri_ + CE-T1WI_peri_ model, and T1WI_intra+peri_ + CE-T1WI_intra+peri_ model all achieved AUC values above 0.90. Of these models, the T1WI_intra+peri_ + CE-T1WI_intra+peri_ model exhibited the highest predictive accuracy (AUC: 0.923, Sensitivity: 86.45%, Specificity: 87.62%, SE: 0.0141). Furthermore, no significant difference in AUC was observed between the T1WI_intra_ + CE-T1WI_intra_ model and the T1WI_peri_ + CE-T1WI_peri_ model (Z = 0.471, *P* = 0.6379). The significance level *P* for all nine models was less than 0.0001.

**Fig. 5:**
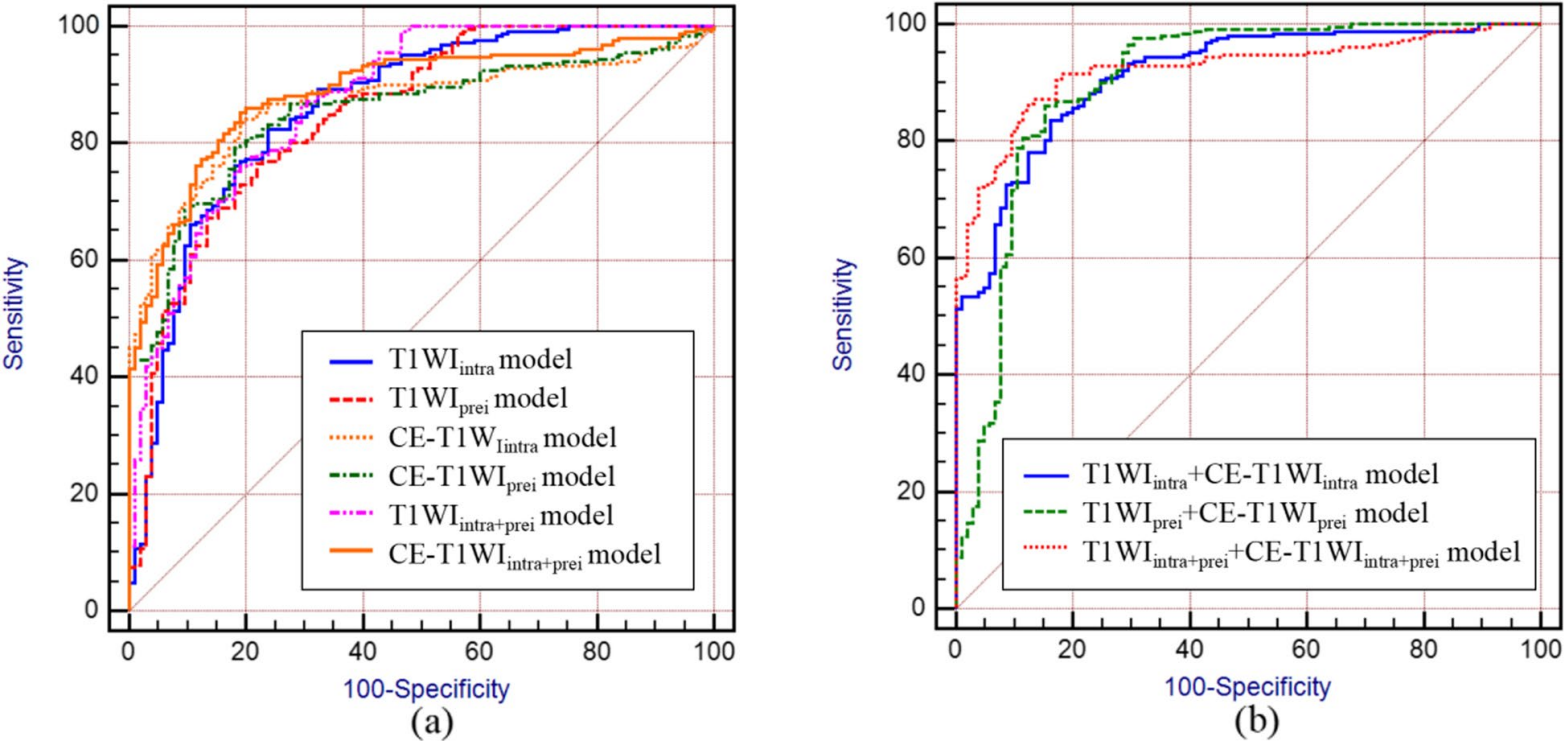
ROC of different models. (**a**) prediction results of single-sequence models, (**b**) prediction results of multiple-sequence models

**Fig. 6:**
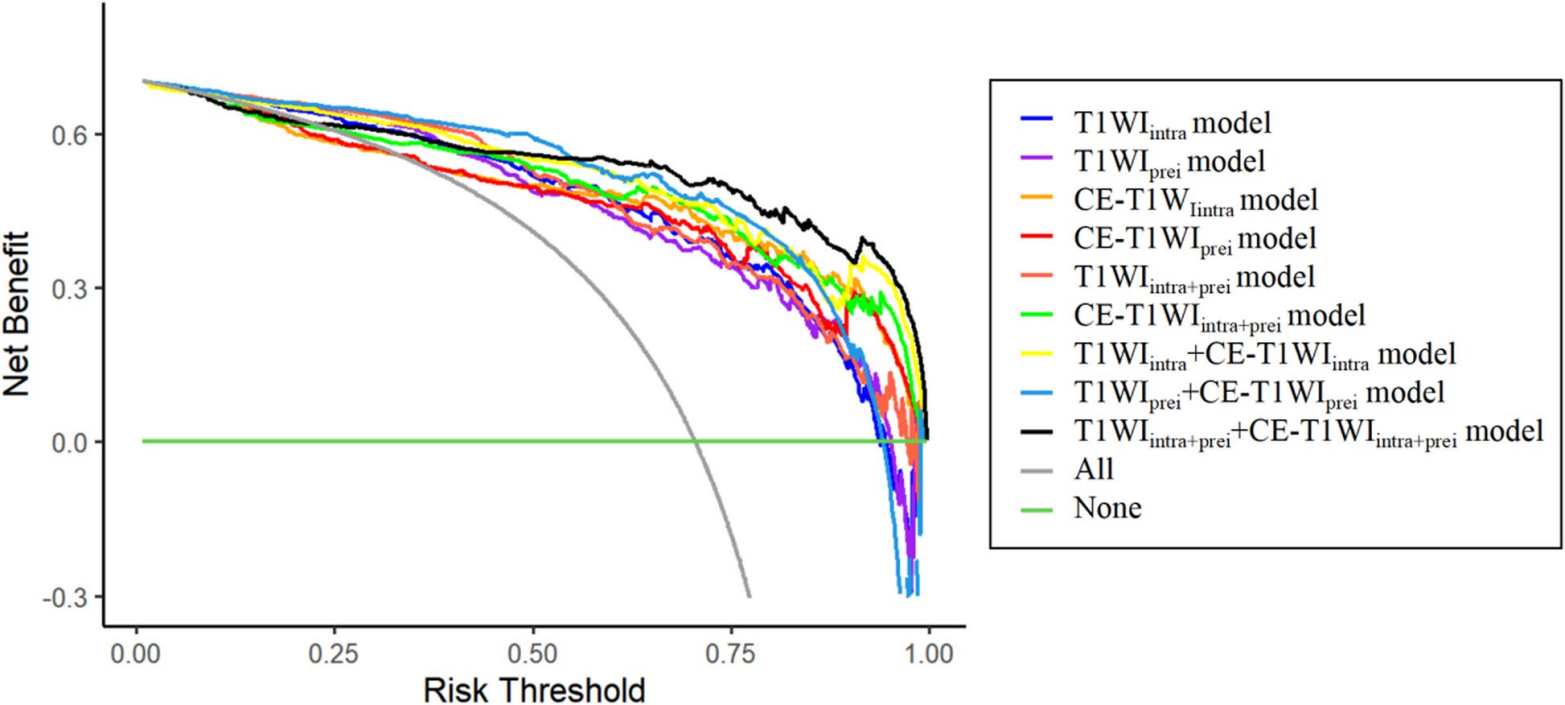
DCA of the different models

**Fig. 7:**
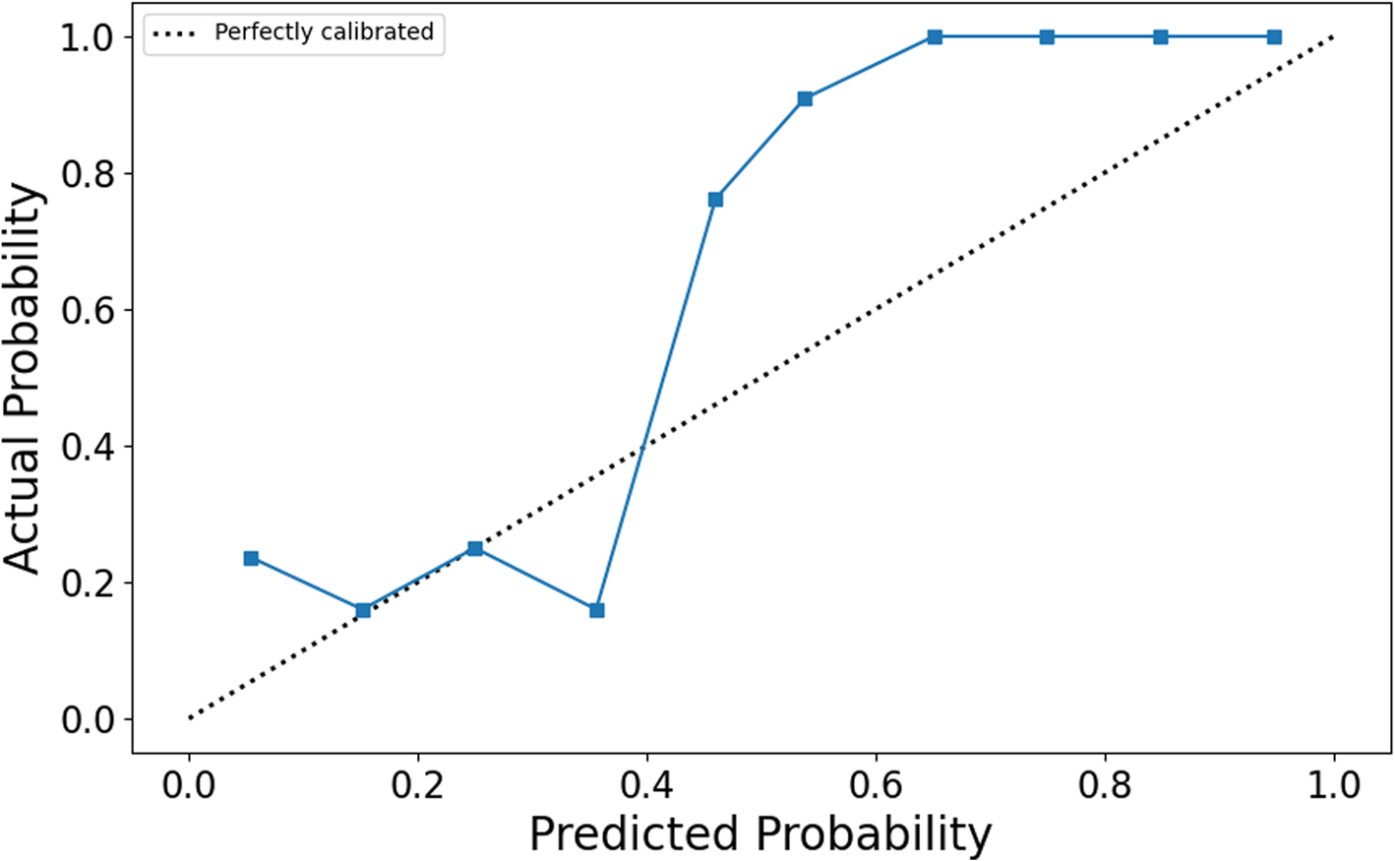
Calibration curve of T1WI_intra+prei_ + CE-T1WI_intra+prei_ model

The ROC curves (Fig. 5) illustrate that models constructed using T1WI and CE-T1WI are effective in diagnosing MGMT promoter methylation status, with all nine models achieving AUC values exceeding 0.85. Based on the H–L test, the *p*-values for the T1WI_intra_ model, T1WI_peri_ model, CE-T1WI_intra_ model, CE-T1WI_peri_ model, T1WI_intra+peri_ model, CE-T1WI_intra+peri_ model, T1WI_intra_ + CE-T1WI_intra_ model, T1WI_peri_ + CE-T1WI_peri_ model, and T1WI_intra+peri_ + CE-T1WI_intra+peri_ model compared to actual observations were 0.115, 0.144, 0.0, 0.0, 0.025, 0.0, 0.0, 0.018, and 0.0, respectively. The latter seven models displayed good agreement with the actual values. Decision curve analysis (DCA) and calibration curve show that the T1WI_intra+peri_ + CE-T1WI_intra+peri_ model was a reliable clinical treatment tool for predicting MGMT promoter methylation status in glioblastoma patients (Figs. 6 and 7).

Moreover, we also compared our method performance with the advanced artificial intelligence method. The 3D-dense-UNet model is used to conduct a comparison due to its applicability in the similar task [23]. We evaluated our proposed method with the 3D-dense-UNet model on the same datasets and under the same experimental conditions to ensure fairness and accuracy. The AUC, sensitivity, and specificity of multi-sequence combined intratumoral and peritumoral model constructed by the 3D-dense-UNet are respectively 84.3%, 85.26%, and 86.67%. In comparison, our model demonstrates an improvement of 8%, 1.19%, and 0.95% in AUC, sensitivity, and specificity over 3D-dense-UNet model, respectively. The comparison results demonstrate that our method performed excellent ability in diagnosing MGMT promoter methylation status.

## Discussion

Establishing relevant prediction models based on the MR image features of the primary tumor using radiomics or machine learning methods have been recognized by numerous studies as an effective method for assessing the MGMT promotor methylation status in glioblastoma patients. For example, a study by Pease et al. showed that a radiomics model based on CE-T1WI and pre-contrast axial T2-weighted Fluid-Attenuated Inversion Recovery (FLAIR) features for predicting MGMT status in glioblastoma, with AUC of 0.85 by leave-one-out-cross-validation [24]. Doniselli et al. constructed a radiomics prediction model using T1WI and 3D FLAIR features of 277 glioblastoma cases, and the results showed that this model can provide a valuable reference for clinical decision-making [25]. Meanwhile, with the popularity of artificial intelligence, predictive models based on deep learning features are increasingly used in clinical practice. Yogananda et al. designed a 3D-dense-UNet deep learning network for the automatic diagnosis of MGMT promoter methylation status on the T2 weighted image dataset of 240 glioma patients [23]. Inspired by this, the present study was the first to use a deep learning approach to extract the T1WI and CE-T1WI features of intratumoral and peritumoral lesions in patients with glioblastoma and to develop the prediction model for assessing the MGMT promoter methylation status using Transformer algorithm. The results showed that these prediction models could effectively distinguish glioblastoma patients in the MGMT promoter methylated group from the unmethylated group, with AUCs of 0.861, 0.850, 0.867, 0.852, 0.874, 0.889 in the T1WI_intra_ model, T1WI_prei_ model, CE-T1WI_intra_ model, CE-T1WI_prei_ model, T1WI_intra+prei_ model, CE-T1WI_intra+prei_ model respectively, indicating that MRI deep learning could be used for the assessment of MGMT promoter methylation status in glioblastoma patients. The findings suggest that models constructed using CE-T1WI outperform those based on T1WI in diagnostic accuracy. This observation is consistent with existing literature [26]. The primary reason is likely due to CE-T1WI’s ability to enhance lesion visualization via contrast agent injection, yielding clearer tumor boundaries and heightened sensitivity to features like intratumoral cystic necrosis and peripheral ring enhancement. These attributes are closely associated with MGMT promoter methylation status. Thus, CE-T1WI demonstrates greater sensitivity in diagnosing MGMT promoter methylation status compared to T1WI.

Due to the presence of glioblastoma tissue heterogeneity, it is often difficult for a single imaging method to provide a comprehensive and accurate assessment of tumorous lesions [27]. In the field of artificial intelligence research, previous studies have also shown that a comprehensive model based on the images features of multiple sequence and clinical factors generally played a better role in the diagnosis and evaluation of tumors than a predictive model based on the images features of a particular imaging sequence [17, 18]. Therefore, this study further developed the T1WI_intra_ + CE-T1WI_intra_ model, T1WI_prei_ + CE-T1WI_prei_ model and T1WI_intra+prei_ + CE-T1WI_intra+prei_ model by combining T1WI and CE-T1WI deep learning features, and clinical factors and compared it with the single sequence models. The results showed that the diagnostic efficacy of the T1WI + CE-T1WI joint models were improved to different degrees compared with the six independent models in the dataset. This suggests that the use of multiple sequence model consisting of T1WI, CE-T1WI, and clinical factors to assess the MGMT promoter methylation status of glioblastoma can provide greater benefit to the patients involved.

Significant clinical evidence [28] suggests that the heterogeneity of glioblastoma is not limited to the tumor margins but also involves the peritumoral region, where approximately 90% of patients with glioblastoma experience recurrence [29]. Malik et al. identified significant differences in the peritumoral regions of glioblastoma and low-grade gliomas, indicating that imaging features from the peritumoral area can assist in differentiating these tumor types [12]. However, the existing MRI-based methods mainly focus on the overall tumors and ignore the value-added role of the peritumoral environment in the study of MGMT promoter methylation status predicting. Studies have verified that interactions between the intratumoral core and peritumoral areas affect tumor development and progression. Integrating features from both regions provides complementary biological information, thereby enhancing model predictive capabilities [30]. The experimental results of this study show that models constructed with combined intratumoral and peritumoral data from T1WI and CE-T1WI sequences consistently outperform those based on either region alone. This is likely due to the significant heterogeneity in glioblastoma within the ET, NCR, and PED regions. The PED region offers additional insights into tumor growth, infiltration, and interactions with normal tissue, which are reflected in specific MRI imaging features, potentially indicating the MGMT promoter methylation status.

Several studies have employed support vector machines [24], random forests [25], and Unet [23] to diagnose MGMT promoter methylation status, achieving notable diagnostic performance. This study is the first to implement the Transformer algorithm in MGMT research, opening new avenues for molecular studies of glioblastoma. The Transformer algorithm has shown remarkable capabilities in glioma segmentation [31], diagnosis [32], and grading [33]. Compared to other methods, the advantage of the Transformer algorithm in medical image processing lies in its robust global context modeling ability, facilitating lesion localization across entire image sequences. Additionally, the Transformer algorithm supports efficient feature extraction and multi-modal information fusion, thereby enhancing model diagnostic performance. Furthermore, given the significant data imbalance for this classification problem, with the ratio of MGMT promoter methylated to unmethylated samples nearing 2.5:1, a dynamically adjusted focal loss was specifically designed as the optimization objective function to counteract the impact of this imbalance on model training. As shown in Table 2, all nine models achieved sensitivity and specificity above 75.30% in predicting MGMT promoter methylation status. Notably, the T1WI_intra_ + CE-T1WI_intra_ model, T1WI_peri_ + CE-T1WI_peri_ model, and T1WI_intra+peri_ + CE-T1WI_intra+peri_ model demonstrated very close predictive results in terms of sensitivity and specificity, all exceeding 83.67%, indicating that the proposed method in this study exhibits excellent accuracy, sensitivity, and specificity in predicting MGMT promoter methylation status. The calibration curve (Fig. 7) suggests good consistency between predicted and observed values for the T1WI_intra+peri_ + CE-T1WI_intra+peri_ model.

Our study has several limitations. First, deep learning studies require large amounts of data and the relative number of subjects with MGMT promoter methylation is small in the TCIA database. Collaborating with other healthcare organizations and research teams will help construct a more representative dataset, enhancing the model's ability to predict MGMT promoter methylation status for glioblastoma. Second, segmenting regions of tumor tissue with similar microenvironments will help analyze the heterogeneity of the lesions. Habitat analysis is a beneficial tool for further exploring and interpreting lesions. Third, biomechanical and biological mathematical models are effective tools for simulating tumor growth and its interaction with heterogeneous tissues. We will attempt to combine imaging data with biomechanical testing (such as stiffness measurements) to more comprehensively describe the physical characteristics of the tumor. Also, other glioblastoma molecules with deep learning features will be studied in our future study.

## Conclusion

In conclusion, deep learning features of glioblastoma, especially the combination of T1WI and CE-T1WI, could reflect tumor molecular pathology indicators of MGMT methylation status. Our model demonstrated high accuracy in diagnosing MGMT promoter methylation status approaching tissue-level performance. This non-invasive marker can facilitate more informed patient counseling and aid in the treatment decision-making process for a significant proportion of patients with glioblastoma.

## Data Availability

The datasets used and analyzed during the current study are available from the corresponding author on reasonable request.

## Abbreviations

MRI: Magnetic resonance imaging
MGMT: O^6^-methylguanine-DNA methyltransferase
TCIA: The Cancer Imaging Archive
T1WI: T1-weighted imaging
CE-T1WI: Contrast-enhanced T1-weighted imaging
ROI: Regions of interest
NCR: Necrotic tumor core
ET: Enhancing tumor
PED: Peritumoral edema
AUC: Area under the receiver operating characteristic curve
TMZ: Temozolomide
AI: Artificial intelligence
MSA: Multi-head self-attention
MLP: Multilayer perceptron
SA: Self-attention
DCA: Decision curve analysis
FLAIR: Fluid-Attenuated Inversion Recovery
